# Cardiac Iodine-123-Meta-Iodo-Benzylguanidine Uptake in Carotid Sinus Hypersensitivity

**DOI:** 10.1371/journal.pone.0126241

**Published:** 2015-06-09

**Authors:** Maw Pin Tan, Alan Murray, Terry Hawkins, Thomas J. Chadwick, Simon R. J. Kerr, Steve W. Parry

**Affiliations:** 1 Ageing and Age-Associated Disorders Research Group, Faculty of Medicine, University of Malaya, Kuala Lumpur, Malaysia; 2 Regional Medical Physics Department, Freeman Hospital, Newcastle upon Tyne, United Kingdom; 3 Institute of Health and Society, Newcastle University, Newcastle upon Tyne, United Kingdom; 4 Institute of Cellular Medicine, Newcastle University, Newcastle upon Tyne, United Kingdom; 5 Falls and Syncope Service, Royal Victoria Infirmary, Newcastle upon Tyne, United Kingdom; University of Otago, NEW ZEALAND

## Abstract

**Background:**

Carotid sinus syndrome is the association of carotid sinus hypersensitivity with syncope, unexplained falls and drop attacks in generally older people. We evaluated cardiac sympathetic innervation in this disorder in individuals with carotid sinus syndrome, asymptomatic carotid sinus hypersensitivity and controls without carotid sinus hypersensitivity.

**Methods:**

Consecutive patients diagnosed with carotid sinus syndrome at a specialist falls and syncope unit were recruited. Asymptomatic carotid sinus hypersensitivity and non-carotid sinus hypersensitivity control participants recruited from a community-dwelling cohort. Cardiac sympathetic innervation was determined using Iodine-123-metaiodobenzylguanidine (123-I-MIBG) scanning. Heart to mediastinal uptake ratio (H:M) were determined for early and late uptake on planar scintigraphy at 20 minutes and 3 hours following intravenous injection of 123-I-MIBG.

**Results:**

Forty-two subjects: carotid sinus syndrome (n = 21), asymptomatic carotid sinus hypersensitivity (n = 12) and no carotid sinus hypersensitivity (n = 9) were included. Compared to the non- carotid sinus hypersensitivity control group, the carotid sinus syndrome group had significantly higher early H:M (estimated mean difference, B = 0.40; 95% confidence interval, CI = 0.13 to 0.67, p = 0.005) and late H:M (B = 0.32; 95%CI = 0.03 to 0.62, p = 0.032). There was, however, no significant difference in early H:M (p = 0.326) or late H:M (p = 0.351) between the asymptomatic carotid sinus hypersensitivity group and non- carotid sinus hypersensitivity controls.

**Conclusions:**

Cardiac sympathetic neuronal activity is increased relative to age-matched controls in individuals with carotid sinus syndrome but not those with asymptomatic carotid sinus hypersensitivity. Blood pressure and heart rate measurements alone may therefore represent an over simplification in the assessment for carotid sinus syndrome and the relative increase in cardiac sympathetic innervation provides additional clues to understanding the mechanisms behind the symptomatic presentation of carotid sinus hypersensitivity.

## Introduction

Carotid sinus hypersensitivity (CSH) may be associated with syncope in older people, and has also been implicated in the etiology of unexplained falls and drop attack [1,2]. Asymptomatic CSH is also known to occur, and is more common than previously anticipated [3], raising the possibility that the exaggerated hemodynamic responses to carotid sinus massage are merely age-related changes rather than CSH being a genuine pathological disorder. Previous work from our group has highlighted altered cerebral autoregulation in patients with carotid sinus syndrome (CSS) compared to control subjects, though the ultimate substrate for this process remains obscure [4]. One candidate may be differences in autonomic function between such individuals. A recent report by our group shows that CSS is associated with enhanced resting sympathetic tone and baroreflex sensitivity [5], with similar results reported from observations of Meyer wave activity in the vasodepressor subtype of CSS [6]. In addition, neuropathological studies in patients with CSS have shown a high burden of hyperphosphorylated Tau protein in medullary autonomic nuclei associated with baroreflex activity [7].

We hypothesize that autonomic function is altered in patients with CSS, and that cardiac sympathetic innervation is enhanced in such individuals. In the study reported here, cardiac meta-iodo-benzylguanidine (MIBG) imaging was used to provide a quantitative measure of cardiac sympathetic innervation. This method is particularly useful in older individuals, as unlike other methods using challenge manoeuvres, it requires minimal patient cooperation. We are aware of no such imaging study in CSS. We, therefore, conducted a case-control study of cardiac MIBG imaging in both patients with CSS, asymptomatic CSH and asymptomatic control subjects without CSH.

## Methods

### Ethics statement

This study was granted ethical approval by the Newcastle and North Tyneside Local Research and Ethics Committee 2. Written informed consent was obtained from all participants. The study protocol conforms to the ethical guidelines of the 1975 Declaration of Helsinki.

### Participants

Participants with CSS were recruited from consecutive patients diagnosed with CSH during investigations for syncope, unexplained falls or drop attacks at a specialist syncope facility. Control participants with and without CSH were recruited from an asymptomatic community cohort evaluated with carotid sinus massage in an epidemiological study [3]. Potential participants were excluded if they were unable to provide informed consent, had a history of thyroid disorders or diabetes mellitus; were taking tricyclic antidepressants or other sympathomimetics; and if there was established ischemic heart disease or clinical features suggestive of heart disease.

### Carotid sinus massage

Carotid sinus hypersensitivity was defined as at least three seconds of asystole or a reduction in systolic blood pressure of at least 50mmHg in response to carotid sinus massage. Carotid sinus *syndrome* was diagnosed if such a response coincided with symptoms reproducing the patient’s original presentation in the absence of a competing cause for such symptoms following comprehensive evaluation in accordance with European Society of Cardiology guidelines [8]. Carotid sinus massage was conducted during continuous electrocardiography and beat-to-beat blood pressure monitoring (Taskforce, CNSystems, Austria) in a bilateral sequential fashion. The position of the carotid sinus was determined by the area of maximal pulsation of the carotid artery, two finger-breadths below of the angle of the mandible, lateral to the thyroid cartilage. Five seconds of longitudinal massage was first performed on the right followed by the left with the patient in the supine position. If no positive response was achieved during supine carotid sinus massage, the procedure was repeated with the patient in an upright position, at a 70° tilt-angle on a tilt-table with a footplate [9].

### Cardiac meta-iodo-benzylguanidine scan

The patients and asymptomatic community dwellers with and without CSH who consented to the study were invited to attend the Medical Physics Department on a separate occasion. Two doses of potassium iodate 170mg were administered in the preceding 24 hours for thyroid blockade. Participants were injected with 150 MBq of 123-iodine radiolabelled MIBG (123-I-MIBG) administered through a peripheral vein. Early (20 minutes) and delayed (3 hours) planar thoracic scintigraphic images were obtained in an anterior projection using a large field gamma camera fitted with a low energy high-resolution collimator. Static images were collected for 15 minutes with a 20% window centred on the 159keV, 123-I photopeak.

**Fig 1:**
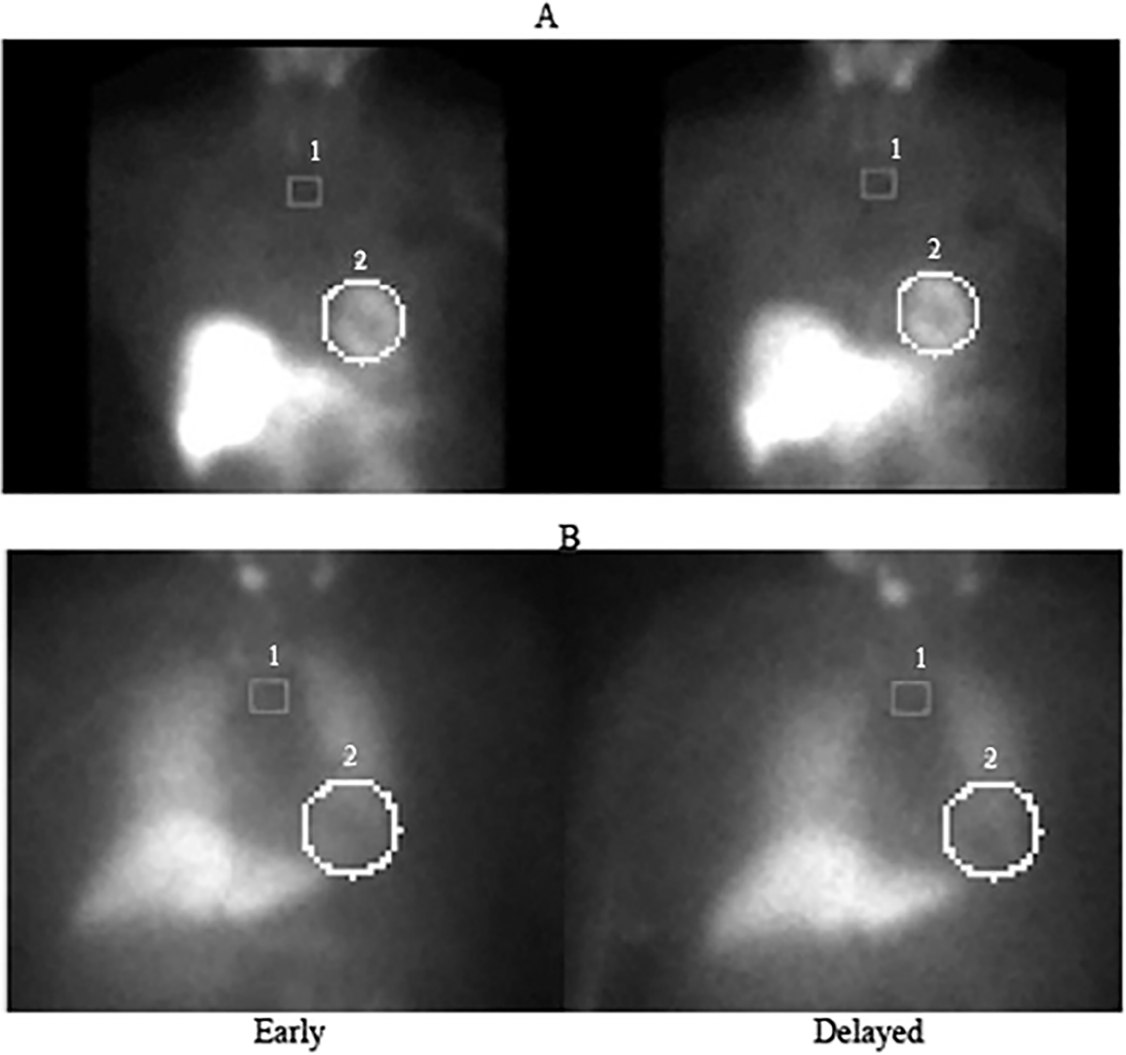
Early and delayed myocardial planar scintigraphy of (A) a participant with symptomatic CSS and (B) an asymptomatic control participant without CSH. The circles (2) highlight the regions of interest for the heart and the squares (1) highlight the mediastinal reference region.

On the early anterior view, an oval shaped region of interest was drawn tightly and semi automatically around the entire heart avoiding as far as possible any confounding activity from adjacent left lung (Fig 1). A small square reference region of interest was drawn in the upper mediastinum between the lung apices [10]. Relative uptake by the heart was determined using the heart to mediastinal (H:M) ratio using the average counts per pixel of the ventricular and mediastinal ROI’s for both early and delayed images. The percentage washout rate (%WR) was calculated using the formula: %WR = [(H:M)early–(H:M)late]**/** (H:M)early * 100. In all cases, late H and M uptake values were corrected for 3 hours of radioactive decay. Ten randomly selected cases were reanalyzed by the same observer and an independent observer to test intraobserver and interobserver agreement. Both observers followed a standardised method of analysis and were blinded to the groups of the study.

### Data analysis

Histogram plots and the Komolgorov-Smirnov test were used to confirm that continuous variables were normally distributed. Continuous variables were then expressed as mean with 95% confidence intervals, while categorical data were presented as count with percentages in parentheses. Participant characteristics were compared with analysis of variance for continuous variables and the Chi-squared test for categorical variables. Interobserver and intraobserver reliability were determined with Pearson’s correlation. Comparisons for early H:M ratio, late H:M ratio and washout rate were made between the CSS group versus non-CSH controls and the asymptomatic CSH group versus non-CSH controls using linear regression methods, based on our hypothesis generated *a priori* that CSH is associated with changes in cardiac sympathetic innervation. Stepwise entry was used for the potential confounders of age, sex, co-existing medical conditions and medications. The B coefficient therefore represents the estimated mean differences between groups. A two-tailed p-value of < 0.05 was considered statistically significant. All statistical analyses were performed using the SPSS™ 15.0 software package.

## Results

### Participant characteristics

Forty-two participants mean age 74 years (range 52 to 90 years) were recruited, 21with CSS, 12 with asymptomatic CSH and 9 asymptomatic controls from the same community cohort who did not have CSH. The characteristics of participants in the above three groups are listed in Table 1.There was a significant difference in the use of lipid-lowering drugs between the three groups (p<0.05), but no other significant differences were present for the remaining characteristics.

**Table 1:** Characteristics of Study Participants.

Characteristics	CSS (n = 21)	Asymptomatic CSH (n = 12)	Non-CSH controls (n = 9)	p-value^a^
Age (Years), mean ± SD	72 ± 10	77 ± 4	74 ± 3	0.248^b^
Male Gender, n(%)	9 (43)	10 (83)	5 (56)	0.077
CSH classification, n(%)				
Cardioinhibitory	11 (52)	6 (50)		
Vasodepressor	10 (48)	6 (50)		
Resting HR (bpm), mean ± SD	73.4 ± 11.5	65.9 ± 13.9	60.0 ± 4.8	0.027
Min HR (bpm), mean ± SD	27.6 ± 19.7	26.4 ± 17.9	48.4 ± 12.7	0.031
Max SBP drop (mmHg), mean ± SD	60.5 ± 11.9	60.3 ± 14.5	33.4 ± 11.4	<0.001
Medical History, n(%)				
Atrial Fibrillation	1 (5)	1 (8)	1 (11)	0.811
Osteoarthritis	5 (24)	6 (50)	4 (44)	0.264
Asthma/COPD	4 (19)	2 (17)	2 (22)	0.950
Cerebrovascular disease	2 (10)	1 (8)	1 (11)	0.977
Hypertension	9 (43)	5 (42)	4 (44)	0.992
Osteoporosis	2 (10)	0	3 (33)	0.059
Medications, n(%)				
Antiplatelets/ anticoagulants	8 (38)	3 (25)	0	0.093
Antipsychotics	2 (10)	2 (17)	0	0.437
Antidepressants	2 (10)	0	1 (11)	0.518
α-adrenoceptor antagonists	1 (5)	0	1 (11)	0.497
β-blockers	1 (5)	0	2 (22)	0.123
ACE- inhibitor	6 (29)	0	1 (11)	0.093
Calcium-channel antagonists,	4 (19)	2 (17)	2 (22)	0.950
Diuretics	4 (19)	1 (8)	3 (33)	0.353
Lipid-lowering agents	9 (43)	2 (17)	0	0.034
Steroids	3 (14)	0	1 (11)	0.398
Proton-pump inhibitors	5 (24)	2 (17)	0	0.276

CSS:carotid sinus syndrome; CSH:carotid sinus hypersensitivity; SD:standard deviation; HR:heart rate; SBP: systolic blood pressure; COPD:chronic obstructive pulmonary disease; ACE:angiotensin-converting enzyme.

a χ^2^-test unless indicated otherwise.

b Analysis of variance.

### Univariate analyses

**Fig 2:**
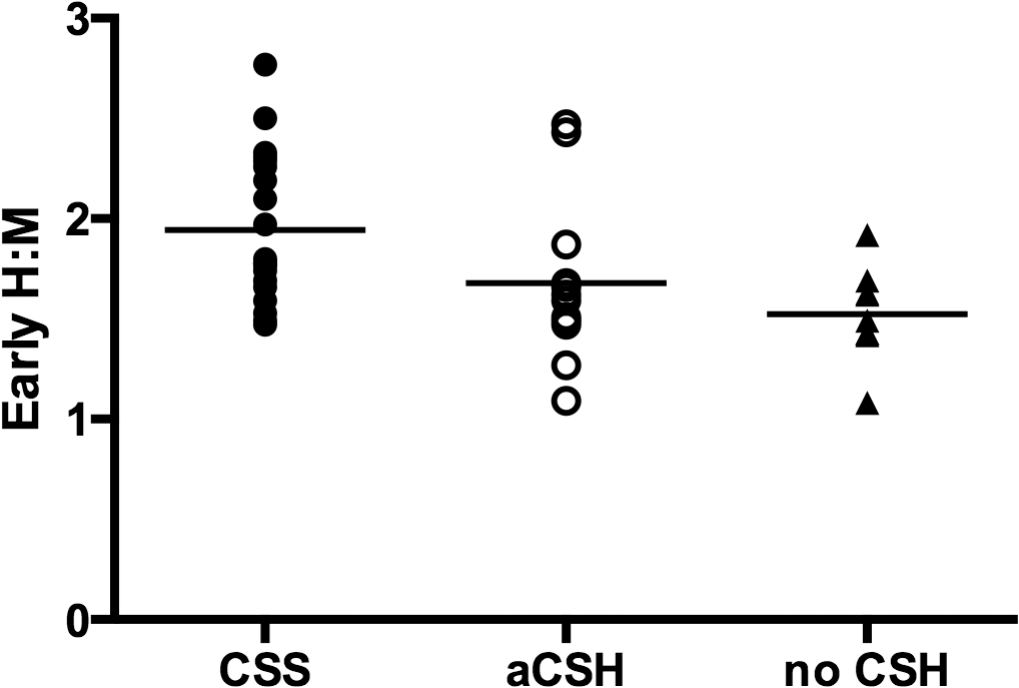
Early Heart to Mediastinal Uptake Ratios. Filled circles, empty circles, and triangles represent individual values, while horizontal lines represent mean values. H:M, heart to mediastinal uptake ratio; CSS, carotid sinus syndrome; aCSH, asymptomatic carotid sinus hypersensitivity; no CSH, no carotid sinus hypersensitivity.

**Fig 3:**
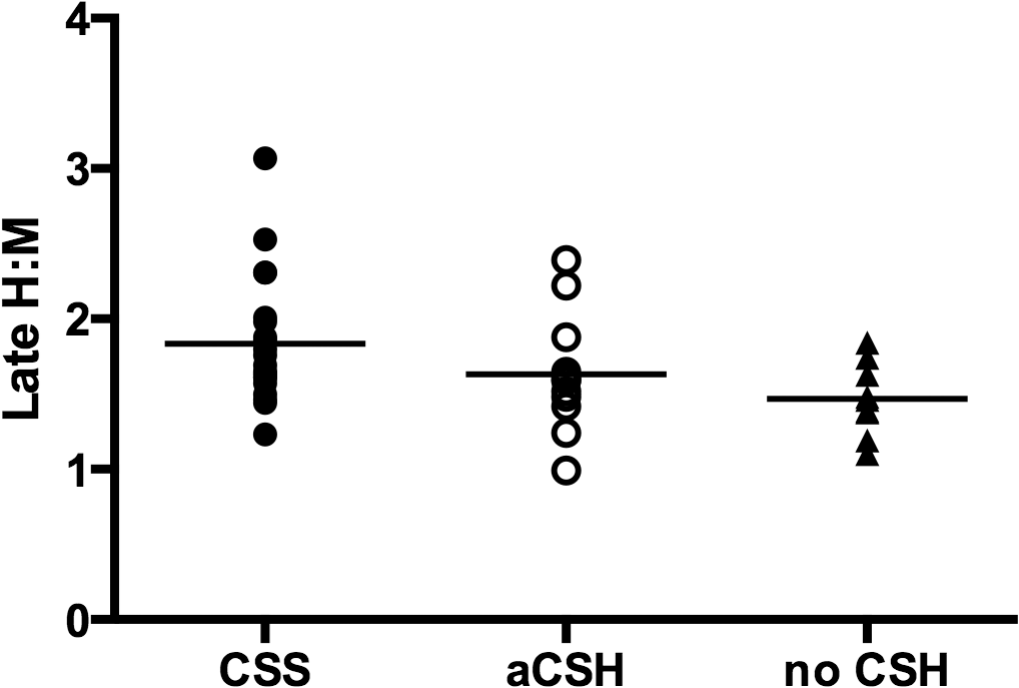
Late Heart to Mediastinal Uptake Ratios. Filled circles, empty circles, and triangles represent individual values, while horizontal lines represent mean values. **H:M**, heart to mediastinal uptake ratio; **CSS**, carotid sinus syndrome; **aCSH**, asymptomatic carotid sinus hypersensitivity; **no CSH**, no carotid sinus hypersensitivity.Symbols represent individual values, while horizontal lines represent mean values.

The intraobserver and interobserver values for the H:M uptake ratios showed good correlation at r = 0.997 and r = 0.994 respectively. Fig 1 shows the 123-I-MIBG scintigraphs for two participants, an asymptomatic control subject without CSH, and the other with CSS with much higher cardiac MIBG uptake. The early H:M uptake ratios for individuals in the three groups are illustrated in Fig 2, while Fig 3 shows the late H:M uptake ratios for individuals in the three groups. The mean early H:M uptake ratios for the CSS, asymptomatic CSH and non-CSH control groups were 1.91 (95%CI = 1.76 to 2.06), 1.69 (95%CI = 1.44 to 1.94), and 1.51 (95%CI = 1.33 to 1.69) respectively. The mean late H:M ratios for the CSS, asymptomatic CSH and non-CSH control groups were 1.80 (95%CI = 1.62 to 1.98), 1.63 (95%CI = 1.38 to1.87) and 1.47 (95%CI = 1.30 to 1.64) respectively. The mean %WR was 19.2 (95%CI = 15.7 to 22.6)% for the symptomatic CSS group, 16.8(95%CI = 12.8 to 20.9)% for the asymptomatic CSH group and 14.5 (95%CI = 10.5 to 18.5)% for the non-CSH control group.

### Multivariate analyses

Linear regression analyses with stepwise entry for potential confounders revealed that the early H:M uptake ratio in individuals with CSS was significantly higher than non-CSH controls (p = 0.005) (Table 2). The late H:M ratio was also significantly higher in the CSS group compared to the non-CSH control group (p = 0.032). There was, however, no significant difference in early or late H:M ratio between the asymptomatic CSH group compared to the non-CSH control group. There were also no significant differences in decay adjusted %WR between the CSS group and the non-CSH control group, or the asymptomatic CSH group and the non-CSH control group. No confounding variables remained in the final models for all of the above variables.

**Table 2:** Multivariate Regression Analysis Displaying Mean Differences in Early and Late H:M Between Groups Compared to Control Participants.

	CSS (n = 21) vs. non-CSH controls (n = 9)		Asymptomatic CSH (n = 12) vs.non-CSH controls (n = 9)	
	Adjusted MD (95% CI)	p-value	Adjusted MD (95% CI)	p-value
Early H:M	0.40 (0.13, 0.67)	0.005	0.18 (-0.12, 0.47)	0.236
Late H:M	0.32 (0.03, 0.62)	0.032	0.15 (-0.17, 0.48)	0.351
% Washout	4.70 (-0.69, 10.1)	0.086	2.34 (-3.63, 8.31)	0.433

CSS: carotid sinus syndrome; CSH: carotid sinus hypersensitivity; MD: mean difference; CI: confidence interval; H:M: heart to mediastinal MIBG uptake; %WR: percentage washout rate adjusted for decay.

## Discussion

### Major findings

Our study was the first to examine cardiac sympathetic innervation through 123-I-MIBG myocardial scintigraphy in both CSS and asymptomatic CSH, and controls without CSH. The CSS group had higher early and late H:M uptake ratios than the non-CSH control group. There were no differences in either ratio between the asymptomatic CSH group and the non-CSH control group. These findings indicate that sympathetic presynaptic neuronal uptake is enhanced in individuals with CSS but not those with asymptomatic CSH. There was, however, no notable difference in %WR which has been considered a marker of sympathetic activity.

### Relationship with existing literature

123-I-MIBG scanning has previously shown higher H:M ratios in head-up tilt positive paediatric patients with vasovagal syncope [11], though an uncontrolled study in adult vasovagal patients showed more variable regional uptake deficits [12]. A more recent study in 60 patients with vasovagal syncope and 20 controls found lower H:M ratios in the vasovagal patients though with highly variable adrenergic innervation deficits in the left ventricular myocardium [13]. These variable and contradictory results in vasovagal syncope suggest the need for considerably more work in this area before definitive conclusions can be drawn. In contrast, sympathetic neuroimaging using 123-I-MIBG scanning has been used extensively in cardiac risk evaluation, in particular in the prognosis of cardiac failure and related risks of ventricular tachyarrhythmias in such patients [14]. It also has a role in the investigation of the alpha-synucleinopathies, providing a reliable method of identifying patients with Parkinson’s disease versus control subjects and those with multiple systems atrophy [15,16]. Intriguingly, a recent study in 24 patients with Parkinson’s disease, 10 of whom had syncope or pre-syncope compared to 14 controls, found that late phase uptake of MIBG was associated with day-time minimum systolic blood pressure [17], raising the possibility of the identification of patients with Parkinson’s who may be more likely to develop syncope. We have previously shown post-mortem autonomic brain stem nuclei associated alpha-synucleinopathy in a patient with carotid sinus syndrome with no clinical evidence of movement disorder, raising the possibility of a causative mechanism for our current findings and for CSS in general [18].

Control subjects in previous studies had included patients with negative CSM or asymptomatic controls with unknown CSH status. CSH has been associated with sternocleidomastoid denervation [19], increased baroreflex sensitivity [20,21] and paradoxical peripheral vasodilatation [22]. We have recently shown alterations in baroreflex sensitivity and heart rate variability in patients with CSS [5], while others have shown altered Mayer wave activity in patients with CSH [6]. Given these findings, and the observation of brain stem degenerative changes in patients with CSS versus age-matched controls [7], we hypothesised that CSS was part of a more widespread autonomic nervous system disorder. In particular, cardiac sympathetic innervation would differ in patients with CSS versus those with CSH and no symptoms, and controls with demonstrably no CSH and no symptoms. This last is a particular strength of our study–the CSH status of all participants was known in contrast to many of the studies mentioned above.

### Explanation of findings

We have shown that cardiac MIBG uptake is increased in individuals with CSS compared to matched controls without CSH. Meta-iodo-benzylguanidine is an analogue of norepinephrine which is not metabolized in humans, and therefore excreted unchanged. The heart is normally enriched with sympathetic nerve endings which also have a high affinity for MIBG, and this property can be used to perform quantitative assessments of the integrity of cardiac sympathetic nerves [23,24]. These findings are complementary to that demonstrated in the recently published evidence that resting sympathetic activity is increased in individuals with CSH [5]. Meta-iodo-benzylguanidine is preferentially taken up by the active transporter system (uptake-1) at sympathetic nerve terminals [25]. An increase in MIBG uptake may therefore be due to either increased neuronal activity leading to increased active transporter uptake or a higher density of sympathetic neurons. Cardiac MIBG uptake is also known to decrease with age [26]. The enhanced MIBG uptake observed in our CSS participants may also signify a relative preservation of sympathetic cardiac innervation against the effects of aging. This may have occurred as a result of chronic sympathetic activation, which is a feature of aging [27], in the presence of loss of negative feedback. The chronic, unregulated, increase in sympathetic outflow may then result in desensitization of post-synaptic adrenergic receptors manifesting as attenuated sympathetic responses during carotid sinus massage.

The relative increase in MIBG uptake observed in the CSS group over the non-CSH control group was, however, not observed in individuals with asymptomatic CSH. The difference in presynaptic neuronal uptake may not, therefore, necessarily account for the heart rate and blood pressure changes that are recorded, but instead appears to be associated with the clinical symptoms that occur with the measured hemodynamic changes. This raises the possibility, reinforcing previous clinical suspicions, that the current practice of assessing heart rate and systolic blood pressure changes alone represents an over simplistic method of assessing overall physiological responses during clinical investigations to evaluate syncope. In addition, this also indicates that individuals with CSH who develop symptoms of falls or syncope have different autonomic responses to asymptomatic individuals, which suggests that CSS is indeed a pathological condition rather than just a feature of normal aging.

The interpretation of the lack of difference in %WR is unclear in the presence of increased early and late H:M uptake. While the washout rate is considered an index of sympathetic drive [28], washout may, however, also signify increased diffusional uptake and increased clearance from the extravesicular pool in individuals with defective active transporter uptake [29,30]. This together with the lack of standardisation of both the acquisition and the analysis of all 123-I- MIBG studies involving cardiac uptake [27] may also have contributed to the failure to identify a lack of anticipated %WR difference between groups in this study.

### Limitations

While the number of participants involved in our study outnumbers all the previous studies evaluating the pathophysiological changes associated with CSH published so far, the actual number remains relatively small, introducing the possibility of false negative results. The clinical applicability of our study is also limited by the potential for overlap between the three groups. Nevertheless, the presence of increased or even preserved cardiac sympathetic innervation in CSS represents an entirely new finding, which at the very least deserves further evaluation. This may well represent the early steps towards the identification of an additional therapeutic target for this increasingly important condition. While treatment in CSS reduces syncope recurrence to 7% at 1 year, at 5 years 26% have recurrence of symptoms regardless of treatment modality [31], so this is far from an academic issue [5].

## Conclusion

Cardiac MIBG uptake was significantly increased in symptomatic individuals with CSH compared to controls without CSH, with no corresponding increase in asymptomatic individuals with CSH and no apparent changes in %WR. The absence of changes in MIBG uptake in asymptomatic individuals with CSH highlights possible inadequacies of heart rate and blood pressure measurements in assessing the overall response to carotid sinus massage. The symptomatic presentation of CSH, carotid sinus syndrome, however, appears to be associated with heightened cardiac sympathetic neuronal activity. This represents an important development in the search for potential therapeutic targets for CSS which is now urgently required.
